# Reduced vancomycin susceptibility in *Staphylococcus aureus* clinical isolates: a spectrum of less investigated uncertainties

**DOI:** 10.1186/s12879-024-10047-2

**Published:** 2024-10-29

**Authors:** Christine E. Tawfeek, Sally Khattab, Nermine Elmaraghy, Anwar A. Heiba, Wedad M. Nageeb

**Affiliations:** Medical Microbiology and Immunology Department, Faculty of Medicine, Suez Canal University, Ismailia, Egypt

**Keywords:** *Staphylococcus aureus*, MRSA, VRSA, VISA, hVISA, VSSA, Reduced vancomycin susceptibility, Vancomycin, Egypt

## Abstract

**Background:**

*Staphylococcus aureus* clinical isolates with vancomycin MICs of 2 µg/ml have been associated with vancomycin therapeutic failure and the heterogenous vancomycin-intermediate *S. aureus* (hVISA) phenotype. While carriage of *van* genes has usually been associated with higher level of MIC and frank vancomycin resistance, the unrecognized risk of hetero-resistance is frequently underestimated. Methods used for assessing vancomycin susceptibility have also shown different concordance and variable performance and accessibility in routine clinical diagnostics posing a challenge to inform treatment selection in hospital settings.

**Methods:**

A total of 195 clinical samples were obtained among which 100 *S. aureus* isolates were identified. Ninety-six MRSA isolates have been identified using cefoxitin disc and *mec*A gene detection. The *van*A and *van*B genes have been screened for in the studied isolates using conventional PCR amplification. Examination of reduced vancomycin susceptibility has been performed using vancomycin screen agar, Broth Micro Dilution method (BMD), and VITEK2. Blood isolates were screened for hVISA using PAP-AUC method.

**Results:**

Vancomycin screening agar applied to 96 MRSA isolates revealed 16 isolates with reduced vancomycin susceptibility. Further MIC testing revealed that 7 isolates were VISA and only 1 isolate was identified as VRSA using both BMD MIC method and VITEK2. Among 24 tested blood isolates, 4 isolates (16.7%) revealed the hVISA phenotype as identified using PAP-AUC method. Using PCR, *van*A gene was identified in 5 *S. aureus* isolates (5%). Three of them were VSSA while the other two isolates were VISA.

**Conclusion:**

In this study, we report the very low prevalence of VRSA among the tested *S. aureus* clinical isolates (1%) and the existence of hVISA phenotype among studied *S. aureus* blood isolates at the rate of 16.7% in our setting. Fifty percent (8/16) of isolates that demonstrated reduced vancomycin susceptibility using vancomycin agar screen tested susceptible using both broth dilution method and VITEK2. These finding together with the concerning silent carriage of *van*A gene among VSSA and VISA (5%) may underly hidden and uninvestigated factors contributing to vancomycin treatment failure that warrant cautious vancomycin prescription.

**Supplementary Information:**

The online version contains supplementary material available at 10.1186/s12879-024-10047-2.

## Background

Methicillin-resistant Staphylococcus aureus (MRSA) has emerged as the most predominant *S. aureus* clones causing clinical infections over the past few decades and by the end of 1990, they have been considered as the most frequent causative agents of *S. aureus* disease in both hospitals and communities [1]. Vancomycin has been used successfully for over 50 years for the treatment of *S. aureus* infections and has been considered as the cornerstone in the management of infections caused by MRSA. The first vancomycin-resistant MRSA was identified in 1997 from a surgical wound infection in a Japanese patient who was not responding to long-term vancomycin treatment [2]. Subsequent isolation of Vancomycin Resistant *S. aureus* (VRSA) then followed from all parts of the world confirming that VRSA has emerged as a global issue [3].

In Egypt, different reports have shown variable levels of VRSA prevalence, but overall, the general rates remain to be relatively low. In a study including 631 *S. aureus* clinical isolates collected from 12 hospitals in Egypt in the period between 2005 and 2013, VRSA was not detected at all in either Hospital Acquired- Methicillin Resistant *Staphylococcus. aureus* (HA-MRSA) or Community Acquired Methicillin Resistant *Staphylococcus. aureus* (CA-MRSA) [4]. In another study, VRSA was detected at the rate of 8.8% (10/114) of *S. aureus* isolates from an Egyptian Intensive Care Unit (ICU) [5]. ElSayed et al., [6] showed a prevalence rate of 13.8% of a total 80 studied *S. aureus* clinical isolates collected from outpatient clinics with no carriage detected at all among 120 *S. aureus* isolates obtained from screening nasal swabs [6]. Another recent study screening for vancomycin resistance among MRSA clinical isolates from Egypt has reported vancomycin resistance in 23.62% of the total studied 127 MRSA isolates with 16.67% and 10% carriage rate of *van*A and *van*B genes among VRSA isolates respectively [7]. In another setting, Gohar et al., [8] have detected only 3 VRSA isolates out of 51 studied MRSA isolates (5.8%) [8]. The pooled general resistance rates of MRSA isolates to vancomycin in Egypt has been reported as 9% [95% CI: 6–12] as shown by a recent meta-analysis study [9].

Although rates of VRSA isolates have been increasing in recent years, the prevalence rates of frank phenotypic vancomycin resistance are thought to be comparatively low in different parts of the world ranging between 5% in Asia, 1% in Europe, 4% in America, 3% in South America, and 16% in Africa according to recent meta-analysis studies [3, 10]. Despite the relatively low prevalence of VRSA worldwide in comparison to other antibiotic classes, there remains the hidden and less frequently investigated causes that may underly vancomycin treatment failure. Microbiologic causes of vancomycin treatment failure may involve vancomycin MIC creep, heterogenous Vancomycin Intermediate *S. aureus* (hVISA), hetero-VRSA, or silent resistance genes carriage. In addition, several other factors related to drug dynamics or other host related clinical factors may contribute to treatment failure. Silent carriage of *van*A operon may be underestimated especially that expression of *van*A-type resistance has high biologic cost for the MRSA host, while in the absence of induction, the biological cost remains at minimum [11]. This indicates that the potential for the dissemination of VRSA clinical isolates should not be underestimated. In addition, the potential of vancomycin treatment failure despite phenotypic susceptibility should not also be underestimated. This may involve microbiologic causes related to discrepant in-vivo/in-vitro behavior or may be attributed to other clinical or host related factors.

Despite being considered a relatively early definition, the phenomenon of hetero-VRSA, in which the bacterial growth exhibits subpopulations with different MICs levels reflected as a mixture of susceptibility levels that include both resistant and susceptible colonies within the same population, has been observed. This phenomenon appears to be not sufficiently studied and thus is under reported due to the changing definitions and breakpoints classifications. A group of *S. aureus* infections designated as hetero-VRSA constitutes a pre-cursor stage to VRSA and exhibit vancomycin resistance only after being exposed to vancomycin and have been linked to infections that are refractory to vancomycin treatment [12]. This may lead to a decline in clinical usefulness of vancomycin use in hospitals.

With the changing breakpoint definitions, the concept of hetero-VRSA has evolved into the definitions of VISA and hVISA phenotypes which appears to be an under-investigated point especially in our region.

Infections caused by hVISA carry a special diagnostic and clinical challenge. Such strains demonstrate in vitro susceptibility to vancomycin (MIC < 4 µg/ml) and thus are classified as susceptible by standard clinical laboratory methods. However, these strains contain subpopulations of at least 1 in 10^6^ cells that can grow in the presence of ≥ 4 µg/ml of vancomycin. Strains demonstrating the hVISA phenotype have been linked to vancomycin treatment failure and poorer prognosis [13–15]. Standardized reference methods for susceptibility testing, such as CLSI broth microdilution, agar dilution, and standard E-test methods, fail to detect hVISA where the population analysis profile-area under the curve (PAP-AUC) method is considered the most reliable and reproducible approach but is labor-intensive, costly, and unsuitable for routine use in clinical laboratories [16]. The pooled prevalence of hVISA between January 1997 to August 2014 has been estimated at 6.05% and that for VISA at 3.01% [17].

A more recent meta-analysis has determined the prevalence of hVISA, VISA, and VRSA isolates among all evaluated *S. aureus* isolates at the rates of 4.6%, 1.7%, and 1.5% respectively [3]. While VISA (2.1%) was more common in Asia, the highest prevalence of hVISA (5.2%) and VRSA (3.6%) were observed in the USA based on subgroup analysis from the same study [3].

The ability to accurately and precisely identify vancomycin MIC especially at the values of 1 and 2 and also at the intermediate category classification has important clinical inferences and needs more careful evaluation [18–20]. Although reported in many studies, this point remains under-investigated. Testing vancomycin susceptibility using different methods including different commercial automated systems does not show agreement as evidenced by several studies [18, 21–25]. It is evident that accurate identification of resistance and measurement of vancomycin MIC is essential for treatment choice and antimicrobial stewardship.

Based on that, having a diagnostic method that can inform use of vancomycin and predict its therapeutic success may be challenging because different factors contribute to resistance in addition to the fact that the ability to measure and assess resistance phenotypes still faces different practical and technical challenges. In this study, we aim to investigate the spectrum of reduced vancomycin susceptibility in MRSA isolates from Egypt using different methods and we shed the light on the occurrence of the frequently underreported phenotypes of hVISA and silent VRSA in our region.

## Methods

### Isolation and identification of *S. aureus* isolates

The study was performed in Suez Canal University Hospital which is a tertiary care hospital established with a capacity of 427 beds with an additional 147 beds for organ transplants and specialized surgeries. The hospital serves the population of Canal Region including citizens of the three canal governorates (Port Said, Ismailia, and Suez) and also serves the governorates of North and South Sinai. Over the period between July 2022 to November 2023, a total of 195 clinical samples were obtained, cultured, microbiologically diagnosed, and analyzed. Various clinical specimens were collected under aseptic precautions from different departments in Suez Canal University hospitals including Intensive care units, outpatient clinics, orthopedic department, and surgery departments. One hundred *S. aureus* isolates were identified among those samples after full microbiologic workup.

### Phenotypic and genotypic identification of MRSA isolates

Methicillin resistance was determined using cefoxitin discs (30 µg) (Oxoid, UK) by disc diffusion method as recommended by Clinical and Laboratory Standards Institute (CLSI). The interpretive criteria according to CLSI guidelines for cefoxitin were *S. aureus*, sensitive ≥ 22 mm and resistant ≤ 21 mm [26]. In addition, PCR amplification of methicillin-resistance *(mec*A) gene was also performed in all isolates using primers listed in Table 1.

**Table 1:** Gene-specific primers used for detection of *mec*A, *van*A, and *van*B in *S. aureus* isolates

Target	Sequence (5′ → 3’)	Annealing temp.	Amplicon size (bp)	Reference
*mec*A	F:5′ AGAAGATGGTATGTGGAAGTTAG-3’ R:5′ ATGTATGTGCGATTGTATTGC − 3’	55 °C	584	Moghaddam, et al., [32]
*van*A	F:5′ GGCAAGTCAGGTGAAGATG − 3’ R:5′ ATCAAGCGGTCAATCAGTTC-3’	55 °C	713	Moghaddam, et al., [32]
*van*B	F:5′ TTCGTCCTTTGGCGTAACC − 3’ R:5′ CGGTTTTCCTGTGAAGTTATCC-3’	55 °C	603	This study

### Detection of Vancomycin resistance encoding genes by PCR

Bacterial DNA was extracted from test isolates using spin-column DNA extraction Kit (Applied Biotechnology Co. Ltd, Egypt). The concentration and purity of chromosomal DNA was determined using nanodrop (NanoDrop Technologies, Wilmington, DE, USA). Single conventional PCR was done to detect presence of *van*A and *van*B among all *S. aureus* isolates using SenoQuest labcycler^®^ thermocycler with the primer pairs as described in Table 1.

### PCR reactions and amplifications conditions

The reaction mixture was prepared in a total volume of 25 µl including 5 µl of template DNA, 12.5 ul of 2X ABT Red master mix (Applied Biotechnology Co. Ltd, Egypt), and 20 Pico-moles of both forward and reverse primers. The volume completed with nuclease free water up to 25 µl. Reaction mixtures without a DNA template served as negative controls. For *mec*A gene amplification, an initial denaturation step was done at 94 °C for 5 min, then 10 cycles consisted of: 45 s of denaturation at 94 °C, 45 s of primer annealing at 65 °C, and 90 s of extension at 72 °C were done. This was followed by another 25 cycles of: 94 °C 45 s, 55 °C 45 s, and 72 °C for 90 s. A final extension step was done at 72 °C for 10 min, followed by a hold at 4 °C. For *van*A gene, initial denaturation was performed for 2 min at 94 °C followed by 35 cycles of denaturation for 1 min at 94 °C, annealing for 1 min at 54 °C, and extension for 1 min at 72 °C. The final extension was performed for 10 min at 72 °C. For *van*B gene, an initial denaturation was performed at 94 °C for 5 min followed by 30 cycles of denaturation at 94 °C for 30 s, annealing at 50 °C for 45 s, and extension at 72 °C for 30 s. The final extension was performed at 72 °C for 6 min. PCR amplicons were analyzed by gel electrophoresis (Major Science, Taiwan) in 2% agarose gel in 1 X Tris-Borate-EDTA (TBE) buffer containing 5 µl /mL ethidium bromide at 100 volts for 45 min and then visualized using UV light. Amplicon size (bp) of the tested genes was identified and compared to a100 bp molecular size standard DNA ladder (Applied Biotechnology Co. Ltd, Egypt) [27].

### Phenotypic identification of reduced vancomycin susceptibility among S. aureus isolates

#### BHI- vancomycin screening agar

All *S. aureus* isolates (100 isolates) were inoculated on vancomycin screening agar containing vancomycin at the concentration of 3 µg/ml to detect vancomycin intermediate and resistant strains using agar dilution method. 0.5 McFarland suspension of each isolate was streaked on the agar. The plates were examined carefully after 24 h incubation at 35 °C for > 1 colony or light film of growth, which meant presumptive reduced vancomycin susceptibility [26, 28, 29].

#### Broth Micro Dilution method

Broth Micro Dilution (BMD) was performed to determine the vancomycin MICs of the strains showing growth on vancomycin screening agar according to CLSI guidelines [26]. Briefly, stock solutions of vancomycin were prepared and two-fold serially diluted to test the final concentrations range of 0.5 to 64 µg/ml. The MIC was determined as the lowest concentration of vancomycin that inhibited visible growth of the organism.

#### Identification of Vancomycin MIC using VITEK2

VITEK2 automated system was also used to determine the MICs of the strains growing on vancomycin screening agar and results were compared to those determined using BMD.

#### Modified population analysis profile- area under curve (PAP-AUC)

Blood isolates of *S. aureus* from bacteremia patients (*n* = 24) were examined for detection of hVISA using the PAP-AUC method according to the method described by Harigaya et al., [30].

Briefly, overnight growth culture of the tested clinical isolates and the Mu3 standard isolate suspensions were adjusted to the modified starting inoculum 10^8^ CFU/ml. Cultures were serially diluted to 10^− 2^ and 10^− 6^, and 100 µl of each dilution was plated in quadruplicate on BHI agar containing vancomycin in the concentrations of 0, 0.5, 1, 2, 4, 6 and 8 µg/ml. Colonies were enumerated after 48 h incubation at 35 °C. The number of colonies in the replicates for each vancomycin concentration was counted, and the number of colonies was converted to number of CFU/ml using the appropriate dilution factor. Bacterial colony counts (Log10 CFU/ml) were plotted against the vancomycin concentration (0 to 8 µg/ml) using GraphPad Prism software (San Diego, CA). The plotted data was then used to calculate AUC. The area under the curve (AUC) was calculated for each isolate and divided by the AUC value of the reference strain Mu3. An isolate with an AUC ratio of ≥ 0.9 was classified as hVISA while an isolate with AUC ratio < 0.9 was classified as VSSA [31].

## Results

### Isolation and identification of isolates

In the present study, 100 isolates out of 195 clinical specimens were identified as *S. aureus* by Gram staining, D-mannitol fermentation, catalase and coagulase production. The identified *S. aureus* isolates were collected from different clinical sources including Blood (*n* = 30), urine (*n* = 11), pus (*n* = 26), skin swab (*n* = 2), sputum (*n* = 14), endotracheal aspirate (*n* = 2), bone aspirate (*n* = 14) and peritoneal fluid (*n* = 1).

### Identification of MRSA isolates

A total of 96 *S. aureus* isolates were identified phenotypically as MRSA. The remaining isolates were identified as MSSA. Molecular identification also revealed that all the 96 isolates harbored *mec*A gene. PCR gel showing the *mec*A gene amplified product is shown in S1 Figure.

### Detection of Vancomycin resistance encoding genes by PCR

The *van*A gene was identified in 5 isolates. Description of isolates with positive *van*A gene is shown in Table 2. The *van*B gene was not detected at all among all tested isolates. In our study, *van*A gene was identified in 5 isolates, 3 of which were Vancomycin Susceptible *S. aureus* (VSSA) when tested phenotypically using both broth microdilution method and VITEK2 automated system (Table 2) and 2 were VISA when tested phenotypically using broth microdilution method (Tables 2, 3 and 5). Unexpectedly, the identified VRSA isolate was negative for both *van*A and *van*B genes (Table 5).

**Table 2:** Characteristics of isolates positive for *van*A gene using PCR

Isolate ID	Vancomycin MIC (BMD)	Case description
41	1 µg/ml	A 42-year-old male patient with no history of chronic diseases presenting with CA-MRSA to the outpatient clinic. Pus sample from an abscess was collected and showed MRSA with positive *mec*A and *van*A genes.
72	0.5 µg/ml	A 63-year-old male patient diagnosed with sepsis in ICU department. Blood sample collected showed HA-MRSA positive for both *mec*A and *van*A genes.
98	1 µg/ml	A 54-year-old male patient with history of COPD admitted to internal medicine ward. Sputum sample was collected with the diagnosis of CA-MRSA and isolate was positive for both *mec*A and *van*A genes.
5	4 µg/ml	A 35-year-old female patient with no history of chronic diseases presenting to outpatient clinic with UTI. Urine sample was collected with the diagnosis of CA-MRSA. The isolate was positive for both *mec*A and *van*A genes.
27	4 µg/ml	A 70-year-old male patient admitted to ICU department with the diagnosis of sepsis. Blood samples were collected with the diagnosis of HA-MRSA. The isolate was positive for both *mec*A and *van*A genes.

COPD: Chronic Obstructive Pulmonary Diseases, UTI: Urinary Tract Infection, CA-MRSA: Community Acquired Methicillin Resistant *S. aureus*, HA-MRSA: Hospital Acquired Methicillin Resistant *S. aureus*

PCR gel showing the *van*A gene amplified product is shown in S2 Figure

**Table 3:** Vancomycin measured MIC values using BMD and VITEK2 method for isolates growing on Vancomycin screen agar (3 µg/ml)

Isolate ID	Susceptibility based on MIC (BMD)	Susceptibility based on MIC (VITEK2)	Type of infection	Genotype
1	> 64 µg/ml **(R)**	> 32 µg/ml **(R)**	Sepsis	*van*A-ve / *van*B-ve
5	4 µg/ml **(I)**	2 µg/ml (S)	UTI	***van*A + ve** / *van*B-ve
6	2 µg/ml (S)	1 µg/ml (S)	UTI	*van*A-ve / *van*B-ve
11	2 µg/ml (S)	2 µg/ml (S)	Abscess	*van*A-ve / *van*B-ve
12	4 µg/ml **(I)**	4 µg/ml **(I)**	Pneumonia	*van*A-ve / *van*B-ve
27	4 µg/ml **(I)**	2 µg/ml (S)	Sepsis	***van*A + ve** / *van*B-ve
90	1 µg/ml (S)	1 µg/ml (S)	SSI	*van*A-ve / *van*B-ve
98	1 µg/ml (S)	1 µg/ml (S)	Pneumonia	***van*A + ve** / *van*B-ve
28	4 µg/ml **(I)**	1 µg/ml (S)	Pneumonia	*van*A-ve / *van*B-ve
33	1 µg/ml (S)	1 µg/ml (S)	Sepsis	*van*A-ve / *van*B-ve
35	4 µg/ml **(I)**	2 µg/ml (S)	Osteomyelitis	*van*A-ve / *van*B-ve
45	2 µg/ml (S)	2 µg/ml (S)	UTI	*van*A-ve / *van*B-ve
81	4 µg/ml **(I)**	4 µg/ml **(I)**	Pneumonia	*van*A-ve / *van*B-ve
83	1 µg/ml (S)	1 µg/ml (S)	Sepsis	*van*A-ve / *van*B-ve
92	4 µg/ml **(I)**	4 µg/ml **(I)**	Sepsis	*van*A-ve / *van*B-ve
100	0.5 µg/ml (S)	1 µg/ml (S)	Osteomyelitis	*van*A-ve / *van*B-ve

BMD: Broth Microdilution Method, (R): Resistant, (S): Susceptible, (I): Intermediate, UTI: Urinary Tract Infection, SSI: Surgical Site Infection, *van*A-ve: Absence of *van*A gene (not detected by PCR), *van*A + ve: Presence of *van*A gene (detected by PCR), *van*B-ve: Absence of *van*B gene

### Identification of MRSA isolates with reduced Vancomycin susceptibility

Out of a total tested 96 MRSA isolates, 16 isolates showed positive growth on vancomycin screen agar (3 µg/ml) and MIC values for those isolates were measured using BMD method and VITEK2. Measured MIC values for these isolates are shown in Table 3. Among these isolates, only 1 isolate was identified as VRSA, 7 isolates were identified as vancomycin intermediate, and 8 isolates were vancomycin susceptible.

For detection of hVISA, bacterial colony counts (Log10 CFU/ml) were plotted against the vancomycin concentration (0 to 8 µg/ml). The area under the curve (AUC) was calculated for each isolate and divided by the AUC value of the reference strain Mu3. An isolate with an AUC ratio of 0.9 was classified as hVISA.

**Fig. 1:**
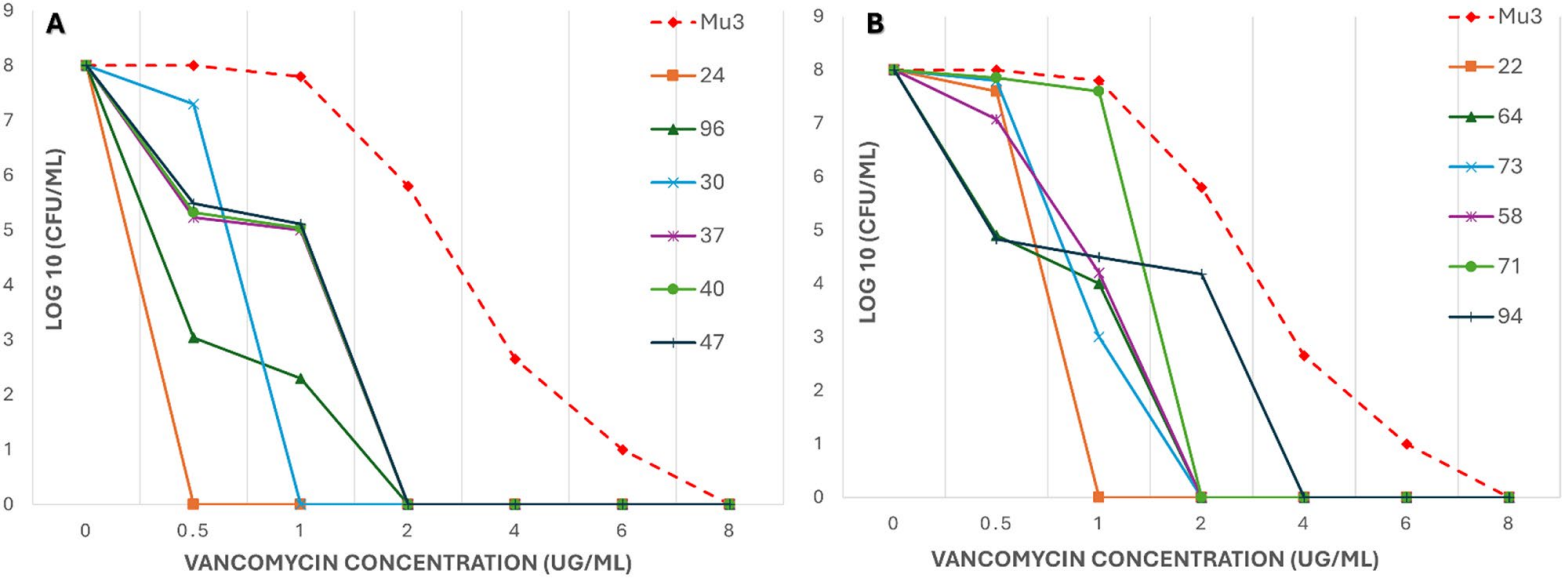
PAP-AUC curves showing tested *S. aureus* blood isolates with lower PAP-AUC ratios

**Fig. 2:**
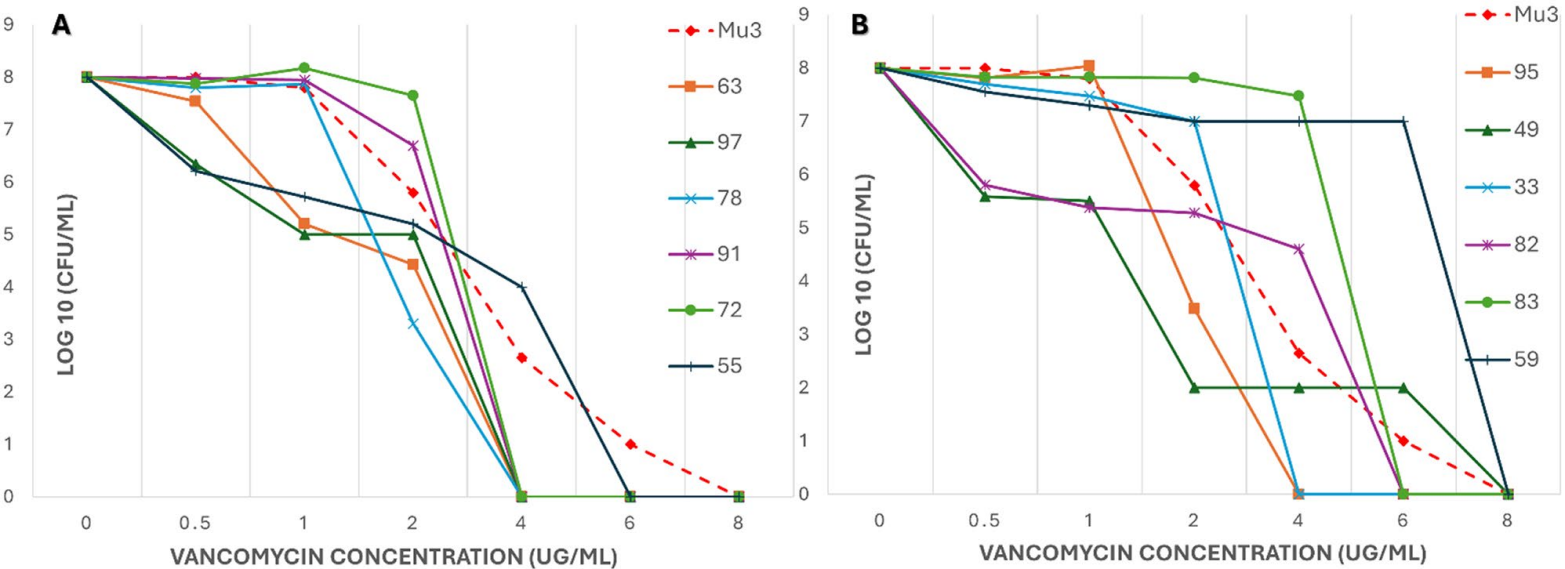
PAP-AUC curves showing tested *S. aureus* blood isolates with higher PAP-AUC ratios

Figures 1 and 2 show curves for tested isolates. Table 4 shows PAP-AUC ratio for tested isolates.

**Table 4:** PAP-AUC ratios for tested *S. aureus* blood isolates

Isolate ID	PAP/AUC ratio	Isolate ID	PAP/AUC ratio
24	0.07	63	0.59
96	0.19	97	0.59
30	0.20	78	0.60
37	0.30	91	0.79
40	0.30	72	0.85
47	0.31	55	**0.90**
22	0.21	95	0.62
64	0.27	49	0.72
73	0.29	33	0.79
58	0.31	82	**0.94**
71	0.42	83	**1.38**
94	0.50	59	**1.79**

Figure 1 presents the isolates with lower AUC ratios (< 0.9) when compared to Mu3 which corresponds with the definition of VSSA. From Figs. 1 and 2, and Table 3, four isolates have demonstrated an AUC ratio ≥ 0.9 which corresponds with the definition of hVISA. These isolates are shown in Fig. 2A and B in dark blue, green, and purple lines.

Table 5 shows the clinical characteristics of VRSA and VISA isolates and their relation to vancomycin susceptibility data. Among the seven VISA isolates, only 2 isolates were positive for *van*A gene.

Growth of each isolate as represented by log10 CFU/ml at different concentrations of vancomycin tested is shown in a different curve color as indicated. Growth of reference Mu3 strain is shown as dashed red line.

**Table 5:** Clinical characteristics of VRSA and VISA isolates

Case number	Vancomycin phenotype classification	Isolate ID	Minimum inhibitory concentration (VITEK 2/BMD)	PCR detection of van A gene	Case clinical description
Case 1	VRSA	1	MIC > 64	*van*A -ve	A 64 old female patient, diabetic with chronic kidney disease. Presented to ICU with coma, dehydration, and sepsis having HA-MRSA and MDR MRSA.
Case 2	VISA	5	MIC = 2/4	***van*** **A + ve**	A 35 old female patient, pregnant with no chronic diseases, presented to outpatient clinic with UTI. CA-MRSA. Resistant to beta-lactams, erythromycin, doxycycline, and clindamycin.
Case 3	VISA	12	MIC = 4	*van*A -ve	Preterm neonate male with pneumonia having HA-MRSA and MDR MRSA.
Case 4	VISA	92	MIC = 4	*van*A -ve	A 25 old male patient, admitted to ICU after accident (trauma) and had sepsis. HA-MRSA isolated from blood sample and MDR MRSA.
Case 5	VISA	35	MIC = 2/4	*van*A -ve	An 8-years-old female patient, presented with osteomyelitis having CA-MRSA and MDR MRSA.
Case 6	VISA	81	MIC = 4	*van*A -ve	Preterm male neonate developed pneumonia in NICU having HA-MRSA resistant to beta-lactams, erythromycin, gentamicin, levofloxacin and ciprofloxacin.
Case 7	VISA	27	MIC = 2/4	***van*** **A + ve**	A 70-years-old male patient, diabetic admitted to ICU with diabetic coma then developed sepsis having HA-MRSA and MDR MRSA.
Case 8	VISA	28	MIC = 1/4	*van*A -ve	Preterm male neonate developed pneumonia in NICU having HA-MRSA resistant to beta-lactam, erythromycin, gentamicin, levofloxacin, ciprofloxacin, and clindamycin.

## Discussion

The first 2 VRSA isolates may have been identified in Egypt in a study including 974 *S. aureus* clinical isolates from Mansoura University Hospitals [33]. The same study has reported the occurrence of hVISA in 27/974 isolates at the rate of 6.08% where 29.63% of them were from blood stream infections. In the same study, VISA was identified in 2.70% of isolates (12/974). Among 117 *S. aureus* isolated from in-patients in an Egyptian hospital in Cairo, vancomycin resistance rate was detected at the rate of 9.5% [34]. In a recent study, 19 VISA (8.8%), and 12 VRSA strains (5.5%) were detected out of 215 MRSA isolates [35].

In our study, we have detected only 1 VRSA isolate among a total of 100 *S. aureus* isolates and 96 MRSA isolates. The identified VRSA isolate was negative for both *van*A and *van*B genes. Like this finding, Tiwari & Sen (2006) have identified 4 VRSA isolates out of a total studied 783 *S. aureus* isolates and none of these isolates have shown *van*A/*van*B gene by PCR [36]. This low prevalence rate may be explained by the presence of 50% CA-MRSA among the studied MRSA isolates. VRSA is believed to occur more frequently in situations where there is expected co-infection or co-colonization of Vancomycin Resistant Enterococci (VRE) with MRSA which is anticipated to happen among patients receiving long-term care and in the intensive care units especially those with indwelling devices, immunocompromised or open wounds which are said to foretell the co-colonization scenario [37]. In addition, patients with VRSA usually demonstrate a history of vancomycin use during the three months before the isolation of VRSA [37]. Larger sample size HA-MRSA may be required in our setting for further study of vancomycin resistance.

VISA showed the prevalence of 7% in our setting. In another study from Egypt, VISA was detected in 1.2% of HA-MRSA (343 isolates) and none was detected in CA-MRSA (21 isolates) [4]. In a study carried out in Menoufia University Hospitals, investigators showed a prevalence rate of 15.2% and 25.3% for VISA and VRSA isolates respectively [38]. VISA infections are reported to be associated with persistent infections, failure of vancomycin treatment, inadequate and poor clinical consequences. In addition, VISA is argued to pose a more threat to the clinic than VRSA as it can evolve in *S. aureus* from different genetic background and is commonly triggered by vancomycin selective pressure that drives its step wise evolution [39].

In our study, 7 isolates were identified as VISA (7%) using BMD while only three isolates were identified as VISA (3%) using VITEK2. Similar to these results, VITEK2 system failed to detect any VISA isolates among 100 MRSA isolates where BMD has detected 22 VISA isolates in the same study [29]. It has been early recognized that VITEK2 system has tendency to under call an MIC of 2 mg/L when compared to BMD [18]. Our study findings support these results. In our study, VITEK2 tended to under call MIC by one-fold dilution except in one isolate in which MIC showed two-fold lower dilution than MIC identified by BMD method. This was similarly observed in previous studies [18]. Another earlier study has also shown that VITEK2 tended to categorize VISA strains as susceptible (undercalling resistance) with some VITEK commercial systems showing MIC results with 2 log_2_ concentrations lower than BMD method [21] which also supports our findings. In another study, Kuo and coworkers, (2020) showed that VITEK2 system tends to overestimate MRSA in high-MIC isolates when compared to the standard BMD method [25]. It has been shown that no method can be considered as absolutely dependable in accurate identification of vancomycin MIC, but BMD method can be considered as the most reliable [40]. Although BMD method is considered the golden standard for MIC determination, it is not being performed in routine clinical diagnostics labs to support treatment prescription by physicians.

The *van*A-type resistance is characterized by high-level activated resistance to both teicoplanin and vancomycin and is mediated by Tn1546 or similar genetic elements [41]. Also, the *van*A gene is acquired on a 100 kb conjugative plasmid transferred from *E. faecium* [42]. Although carriage of *van*A gene is commonly reported as an underlying mechanism of high-level vancomycin resistance, intermediate susceptibility to vancomycin has been previously demonstrated experimentally to be linked to variable expression of the *van*A gene cluster [43], however, this mechanism is not widely reported. Several studies have linked the presence of *van*A gene to the expression of high-level vancomycin resistance with MIC ranging between 32 and 1024 µg/mL [37, 44].

In our study, *van*A gene was identified in 5 isolates, 3 of which were VSSA when tested phenotypically using both broth microdilution method and VITEK2 automated system and 2 were VISA when tested phenotypically using broth microdilution method. This finding underscores the seriousness and an unexpected risk of hetero-VRSA or Silent VRSA. Our results also show that 8 out of 16 (50%) strains that screened positive using vancomycin screen agar tested sensitive using both MIC broth dilution method and VITEK2. This finding also points to the risk of carrying silent resistance elements or expressing vancomycin heteroresistance which may not be commonly detected using routine methods used for susceptibility or MIC testing.

Like our findings, Saadat et al., [45] demonstrated that 19/100 studied *S. aureus* showed either *vanA* or *vanB* carriage but were susceptible according to vancomycin agar screening test [45]. Also, Elkhyat et al., [38] showed the carriage of *van*A gene in a vancomycin susceptible *S. aureus* burn isolate [38].

Previous studies have shown that heterologous expression of the *van*A operon may underly intermediate levels of vancomycin resistance [43, 46]. Glycopeptide resistance in strains containing the *van*A gene cluster has been shown to vary in stability and inducibility, with low levels of resistance ascribed to plasmid instability together with a protracted induction delay for resistance [46]. Additionally, when Glycopeptide resistance stability was tested, results showed that resistance was lost at high frequency after an overnight growth without the antibiotic concluding that Low-level glycopeptide resistance is most likely attributed to the instability of the transposon, plasmid, or a genetic element that carries the *van*A operon and is associated with a lengthier lag phase before growth starts again after being induced by vancomycin [43].

Worries about in vivo switching and horizontal spread of *van*A + vancomycin variable enterococci have been demonstrated by Audun et al., [47] showing the potential of a silenced *van*A gene cluster on a transferable plasmid to cause outbreaks. Authors have shown that a silenced *vanA* that can be horizontally transferred may be able to pass undetected in the routine clinical workflow and can revert into resistance after starting vancomycin therapy representing a new confront in clinical practice. The findings of our study draw a new highlight confirming these findings. Audun et al., [47] have shown that IS*L3*-family element which is found upstream of *vanHAX* can silence the transcription of *vanHAX* in vancomycin variable enterococci (VVE) [47]. Furthermore, the same authors showed that the VVE had IS1542 which can be found inserted between *van*R and orf2 and this is believed to attenuate the expression of *van*HAX. The *vraTSR* operon has also been shown to significantly affect *van*A operon expression where its mutations or loss were associated with a two-to five-fold reduction in the *van*A operon genes’ expression [48].

In 2008, researchers from China have identified hVISA at the rate between 13% and 16% in a survey including 1012 MRSA strains obtained from fourteen cities [49]. A study from Latin America evaluating a total of 1189 vancomycin-susceptible MRSA isolates has recovered a total of 39 MRSA isolates (3.3%) that were classified as hVISA [50]. A study from India has reported an hVISA phenotype at 12.4% among a total 500 clinical isolates of MRSA [51].

The hVISA phenotype has been frequently reported in patients with bacteremia. In a study from USA, PAP/AUC revealed 6 (1.6%) VISA and 30 (8.1%) hVISA phenotypes among a total of 371 MRSA blood isolates [52]. Another study from Australia screening 458 MRSA blood isolates has identified four (1%) VISA isolates and fifty-five (12%) hVISA isolates using PAP-AUC method [53]. Another study has revealed hVISA in 220 clinical *S. aureus* isolates from blood stream infections at the rate of 5.5% which was higher among MRSA isolates (9.1%) [30]. A higher rate was detected in another study from Korea showing that 121/382 patient with MRSA bacteremia (32%) had hVISA bacteremia [15]. A similarly high rate was also detected in a more recent study from Korea showing that about 30% of MRSA strains isolated from blood were identified as hVISA [54]. In Argentina, hVISA was detected in 3 out of 92 patients with *S. aureus* blood isolates [55].

In Egypt, very few studies have evaluated the prevalence of hVISA. A study from Mansoura University Hospitals has detected hVISA at the rate of 6.08% [33]. In Alexandria, investigators detected hVISA at 6/62 studied isolates (9.68%) [56] Another study from Mansoura evaluating a total of 58 coagulase negative Staphylococci (CoNS) has identified Nine isolates (15.5%) as hVISA [57]. Another study including 100 MRSA isolates referred to Central Microbiology Laboratory of Ain Shams University Hospitals has identified hVISA at the rate of 9% [29]. In our study, we identify hVISA at the rate of 4/24 tested blood isolates (16.7%) highlighting the need for careful consideration and assessment of this phenotype further from different clinical samples and from different study settings and geographic localities in Egypt.

## Conclusion

In our study, we highlight that reduced vancomycin susceptibility may originate from hVISA phenotype or due to the silent carriage of *van*A gene which could emerge into hetero-VRSA phenotype, and both are not commonly tested in the routine procedures used to test for vancomycin susceptibility. These factors can unfortunately contribute to vancomycin treatment failure which warrants revising common considerations used for vancomycin prescription.

Although the prevalence of vancomycin heteroresistance is not considered to be high in the study setting, silent vancomycin resistance may be a possible unforeseen contributor to vancomycin therapeutic failure. Many technical and practical challenges still face the recognition and management of vancomycin heteroresistant, intermediate and resistant phenotypes carrying an unrecognized possibility of vancomycin treatment failure. The *vanA* operon, and the *vanB* operon typically carried on the transposable elements Tn1546 and Tn1549 have been reported as the most prevalent globally underlying identified cases of VRSA in *S. aureus* and have been linked to acquired and transmissible vancomycin resistance in *S. aureus*. However, we cannot exclude the presence of other *van* operons which are commonly reported in *Enterococcus* species, and we would recommend further testing for the future occurrence of these operons in *S. aureus* in such cases. Another limitation of the current study is that we could not perform SCCmec genotyping, or ST genetic lineage identification to find their possible link to reduced vancomycin susceptibility phenotypes.

## Electronic supplementary material

Below is the link to the electronic supplementary material.

Supplementary Material 1

## Data Availability

All data generated or analyzed during this study are included in this published article.

## Abbreviations

*S. aureus*: *Staphylococcus aureus*
MRSA: Methicillin Resistant *S. aureus*
HA-MRSA: Hospital-acquired MRSA
CA-MRSA: Community acquired MRSA
hVISA: Heterogenous vancomycin-intermediate *S. aureus*
VRSA: Vancomycin Resistant *S. aureus*
VISA: Vancomycin Intermediate *S. aureus*
VSSA: Vancomycin Susceptible *S. aureus*
ICU: Intensive Care Unit
BMD: Broth Micro Dilution
PAP-AUC: Population Analysis Profile-Area Under Curve
MIC: Minimum Inhibitory Concentration
CLSI: Clinical and Laboratory Standards Institute
UTI: Urinary Tract Infection
SSI: Surgical Site Infection
CoNS: Coagulase Negative Staphylococci
