# Supplementary material for: Reduced vancomycin susceptibility in Staphylococcus aureus clinical isolates: a spectrum of less investigated uncertainties
Source: BMC Infect Dis. 2024 Oct 29;24:1218. doi: 10.1186/s12879-024-10047-2 (PMC11520445; doi:10.1186/s12879-024-10047-2)
Supplement: Supplementary file 1 — Supplementary Material 1 [file 12879_2024_10047_MOESM1_ESM.pdf]

Reduced vancomycin susceptibility in *Staphylococcus aureus* clinical isolates: A spectrum of less investigated uncertainties

Christine E. Tawfeek [1], Sally Khattab [1], Nermine Elmaraghy [1], Anwar A. Heiba [1], Wedad M. Nageeb [1]*

[1] Medical Microbiology and Immunology Department, Faculty of Medicine, Suez Canal University, Ismailia, Egypt

S1 Figure: PCR gel showing the *mec*A gene amplified product

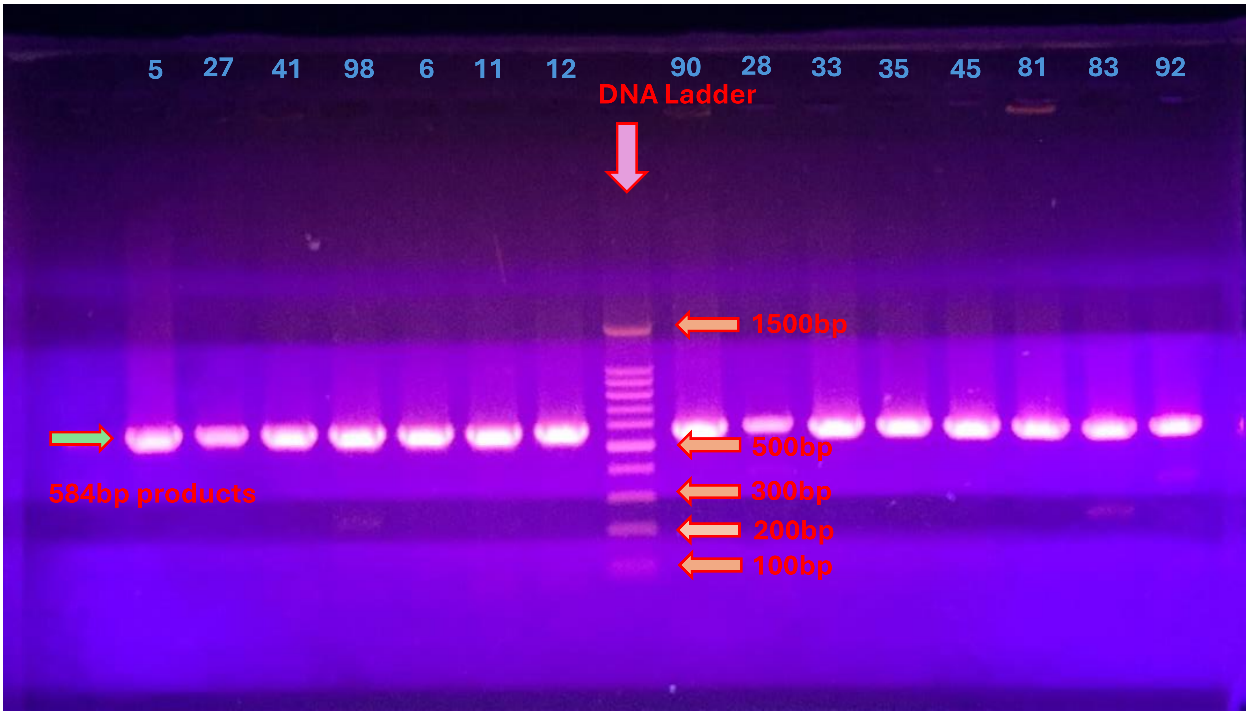

Isolates are shown in lanes with different samples tested, Vertical pink arrow show DNA ladder with size of marker bands shown in orange arrows. Product size of 584 bp positive in all lanes of all samples shown corresponds to *mec*A gene.

S2 Figure: PCR gel showing the *van*A gene amplified product

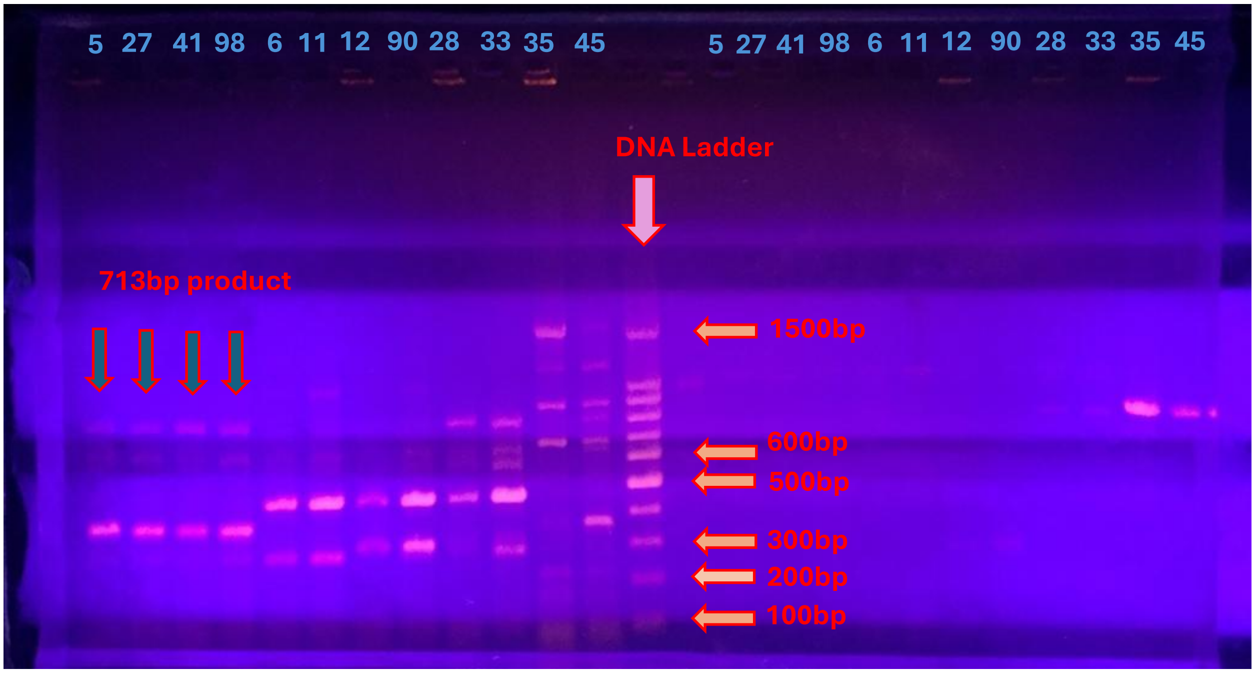

Left Side S2 Figure: Vertical green Arrows show product size of 713bp corresponding to *van*A gene, Vertical pink arrow DNA ladder with size of marker bands shown in orange arrows.

Right Side S2 Figure: All lanes were negative for *van*B gene with no PCR products.
